# Interparental Positivity Spillover Theory: How Parents’ Positive Relational Interactions Influence Children

**DOI:** 10.1177/17456916231220626

**Published:** 2024-01-22

**Authors:** Brian P. Don, Jeffry A. Simpson, Barbara L. Fredrickson, Sara B. Algoe

**Affiliations:** 1 School of Psychology, University of Auckland; 2 Department of Psychology, University of Minnesota; 3 Department of Psychology, University of North Carolina at Chapel Hill

**Keywords:** parent relationships, child development, intimate relationships, positive psychology

## Abstract

Interparental interactions have an important influence on child well-being and development. Yet prior theory and research have primarily focused on interparental conflict as contributing to child maladjustment, which leaves out the critical question of how interparental positive interactions—such as expressed gratitude, capitalization, and shared laughter—may benefit child growth and development. In this article, we integrate theory and research in family, relationship, and affective science to propose a new framework for understanding how the heretofore underexamined positive interparental interactions influence children: interparental positivity spillover theory (IPST). IPST proposes that, distinct from the influence of conflict, interparental positive interactions spill over into children’s experiences in the form of their (a) experience of positive emotions, (b) beneficially altered perceptions of their parents, and (c) emulation of their parents’ positive interpersonal behaviors. This spillover is theorized to promote beneficial cognitive, behavioral, social, and physiological outcomes in children in the short term (i.e., immediately after a specific episode of interparental positivity, or on a given day) as well as cumulatively across time. As a framework, IPST generates a host of novel and testable predictions to guide future research, all of which have important implications for the mental health, well-being, and positive development of children and families.

Consider the familial experience of two adolescent boys: Jamal and Mike. Both Jamal’s and Mike’s parents rarely have conflicts, disagreements, or arguments. However, Jamal’s and Mike’s parents differ on another dimension: the extent to which they engage in *positively valenced interactions*. Jamal’s parents have a relationship in which they frequently have positive interactions. For instance, they often express their appreciation to each other, share good news (and respond positively to their partner’s good news), and share laughter. Mike’s parents, by contrast, rarely engage in positive interactions; for example, they rarely express appreciation, share good news, or laugh together.

This example introduces the central question of this work: What impact do positive interparental interactions have on children? Although prior research has demonstrated that (a) interparental conflict affects children in important ways (Cummings & Miller-Graff, 2015) and (b) positive interactions in intimate relationships contribute to beneficial outcomes for individuals in those relationships (Algoe, 2019) and for nonfamilial third parties who observe these interactions (Algoe et al., 2020), little research has examined the implications of positive interparental interactions on their children. Drawing on theories in family, relationship, and affective science, here we present interparental positivity spillover theory (IPST), which posits that interparental positive interactions play an important and unique role in promoting their children’s well-being and development. In particular, IPST suggests that interparental positive interactions promote beneficial outcomes for children during particular moments, and cumulatively across time, because interparental positive interactions spill over into children’s experiences in the form of (a) increased intensity and frequency of positive emotions, (b) beneficially altered perceptions of their parents, and (c) social learning of positive behaviors. In what follows, we first review prior research and theory and then propose a model detailing how interparental positive interactions can influence their children.

## Emotional Security Theory and the Importance of the Interparental Relationship for Children

One prominent theory of interparental interactions and child adjustment from the developmental science literature is emotional security theory (EST), which suggests that the quality of interparental interactions, in addition to the parent–child bond itself, shapes child well-being and development (Cummings & Miller-Graff, 2015; Davies & Cummings, 1994). Whereas other perspectives, such as attachment theory and developmental psychopathology, emphasize the quality of the direct relationship between parents and their children (Brumariu & Kerns, 2010; Cicchetti & Toth, 2009; Masten, 2006), EST critically and novelly extends these approaches by postulating that the interparental relationship has an additional impact on children that is distinct from the parent–child relationship (Davies & Cummings, 1994; Davies & Martin, 2014; van Eldik et al., 2020). That is, EST claims that what happens in interparental relationships uniquely contributes to child outcomes, independent of the quality of parent–child relationships. Notably, this proposition is consistent with other foundational theories in the family-science literature, such as family-systems theory (Cox & Paley, 2003), which suggests that all relationships within the family system have the potential to influence each individual within the family.

Importantly, the predominant focus of EST is on interparental conflict. That is, EST posits that (a) children have an innate need for safety, security, and protection and (b) interparental conflict threatens this sense of security within the family (Davies & Cummings, 1994; Davies & Martin, 2014). Thus, children’s emotional and behavioral outcomes in response to interparental conflict are believed to reflect their desire to reestablish emotional security, and an extensive body of evidence supports these propositions (Cummings & Miller-Graff, 2015; Davies & Martin, 2014; van Eldik et al., 2020). For instance, interparental conflict predicts a host of important child outcomes, such as greater internalizing symptoms, greater externalizing symptoms, and less social competence (Davies et al., 2016). Indeed, hundreds of studies support the predictions of EST, providing clear evidence that interparental conflict shapes child well-being and development (Cummings & Davies, 2011; van Eldik et al., 2020). For instance, a meta-analysis by van Eldik et al. (2020) quantitatively summarized the literature on the basis of the results of 169 studies, demonstrating that interparental conflict frequency and hostile conflict behavior had small to moderate effect sizes on key child outcomes such as their emotions (e.g., anger, sadness, fear), perceptions of their parents, and internalizing and externalizing behaviors.

Although existing evidence clearly demonstrates that interparental conflict threatens children’s emotional security and can have deleterious effects on their well-being and development, focusing solely on interparental conflict omits a critical part of the familial and developmental picture. Indeed, the strong focus on interparental conflict on child outcomes within the scientific literature mirrors a broader pattern within the developmental-psychology literature: Some scholars have suggested that a “pathology model” has been one of the primary ways in which children have been viewed and studied (Van Allen et al., 2021). Researchers have typically focused on children and families who experience the most challenging or problematic behaviors and outcomes to the relative neglect of understanding the processes that allow children and families to thrive. We adopt a different approach. Although interparental conflict is clearly important, we propose that, through positive interactions, the interparental relationship has the capacity to promote children’s well-being and development in numerous ways.

## Positive Interparental Interactions and Child Well-Being

IPST suggests that positive interactions between parents (couples) can have a unique and beneficial effect on their children’s well-being and development, and that this occurs even while accounting for the direct influence of the parent–child relationship or interparental conflict. Whereas EST outlines the capacity for the interparental relationship to impact children, this body of literature focuses only on a specific, limited range of interparental interactions (i.e., conflict and negative exchanges). Moreover, regarding the child’s needs, EST focuses only on security. Numerous theoretical perspectives, however, posit that addressing only a lack of harm ignores the fundamental human (and child) need to thrive.

Self-determination theory, for instance, suggests that humans possess an organismic capacity to learn, grow, and flourish, and that people require positive, nourishing environments to unlock this organismic potential toward growth and healthy development (Ryan & Deci, 2017). Attachment theory also proposes that, in addition to security (i.e., a safe haven), humans also need nourishing, positive environments (i.e., a secure base) from which growth, exploration, and positive development can occur (Bowlby, 1988; Feeney & Thrush, 2010). Other theories in positive psychology and developmental science concur that focusing only on child security neglects the importance of nourishment or the positive aspects of a family environment that support a child’s innate tendency to thrive (e.g., Masten, 2006; Stifter et al., 2020; Van Allen et al., 2021). Thus, although the evidence is clear that interparental conflict adversely affects a multitude of child outcomes, IPST takes the novel stance that it is critical to consider a wider range of interparental interactions to more fully understand the well-being and development of children. Accordingly, IPST argues that positive interparental interactions should also be considered a vital part of the developmental picture because they can help to create the nourishing family environment that children need to flourish.

What specific types of positive interparental interactions might shape children? Algoe (2019) defined positive interpersonal processes as interactions that are infused with positive emotions in which one individual’s thoughts, feelings, and behaviors affect another person. Prototypical examples include expressions of gratitude (Algoe et al., 2013); sharing good news (capitalization; Gable et al., 2006); shared laughter between relationship partners (Kurtz & Algoe, 2015); moments of coexperienced, kind-hearted amusement; and expressions of admiration for one’s partner. Positive interpersonal processes are distinct from other types of interpersonal processes because they are positively valenced during actual interactions, as opposed to merely contributing to beneficial outcomes. To provide a contrasting example, although social-support interactions in response to negative events in relationships can also generate beneficial outcomes, they are often (although not always) stressful and challenging during the actual interaction (e.g., discussing a stressful problem that involves mixed or negative emotions). Positive interpersonal processes, on the other hand, are distinct because the interaction itself is fueled by pleasant feelings. When an individual shares good news with their partner, for instance, the interaction is driven by the positive emotional state that initially launched the interaction. It is also worth noting that just as the experience of positive emotions and negative emotions typically are not strongly correlated (e.g., Ong et al., 2017), positive and negative interactions within intimate relationships are also typically not strongly correlated (e.g., Zemp et al., 2019). What this means is the degree of conflict within a couple’s relationship is not necessarily the inverse of the level of positivity within their relationship.

Research from several prospective dyadic studies indicates that these types of positive interactions predict a host of beneficial outcomes. Within intimate relationships, for example, expressions of gratitude are associated with enhanced perceptions of partner responsiveness, more positive emotions, and buffer against the negative consequences of attachment insecurity (Algoe et al., 2013, 2016; Park et al., 2019). Interactions in which one partner shares personal good news with their partner (i.e., capitalization interactions) also contribute to beneficial outcomes in close relationships by promoting greater positive affect, more intimacy, and higher relationship satisfaction (e.g., Gable et al., 2006, 2012; Peters et al., 2018; Reis et al., 2010).^1^ Despite this, little prior theory or research has considered the possibility that positive interactions between parents have implications for their children (for exceptions, which we return to later, see Zemp et al., 2014, 2019).^2^

We note here that IPST, like EST, is consistent with the predictions of family-systems theory (Cox & Paley, 2003), according to which families are tightly connected systems in which every individual within a family, and every relationship within the family, influences each other. Thus, according to family-systems theory, the functioning of any individual in a family system depends to some extent on other individuals and interrelationships within the family. IPST’s core predictions—that parents’ positive interactions will spill over to influence the well-being and development of children—fit well within the broader framework of family-systems theory.

## Understanding Interparental Positivity Spillover: The “What” and the “How”

What exactly happens when interparental positive interactions spill over into child experiences? Moreover, how does this spillover result in beneficial child outcomes? First, the “what”: IPST suggests that when parents engage in positive interactions, such interactions spill over into child experiences; that is, they spill over in the sense that the positivity embedded in the interparental interactions results in fundamental changes in the children’s psychological experiences and behavior, even when the children are not directly involved in the initial interaction. IPST posits that interparental positive interactions spill over into children’s experiences in three key ways: the elicitation of positive emotions (Prediction 1a), beneficially altered perceptions of their parents (Prediction 1b), and social learning of positive interpersonal behaviors (Prediction 1c).^3^ According to IPST, these three subcomponents collectively constitute interparental positivity spillover.

Second, the “how”: According to IPST, these three subcomponents of spillover are the primary mediators for the beneficial child outcomes created by interparental positive interactions. That is, by generating positive emotions in children, altering children’s perceptions of their parents, and modeling positive interpersonal behaviors for children, interparental positive interactions contribute to important child outcomes. This can result from individual interparental positive interactions (i.e., in the short term) or from accumulated interactions across time. In the next section, we further discuss the ways in which interparental positive interactions are likely to manifest in these three subcomponents of spillover. Then, in the following section, we describe how the specific subcomponents of spillover likely mediate the influence of interparental positive interactions on beneficial outcomes for children.

## What Is Interparental Positivity Spillover? Explicating the Three Subcomponents

### Spillover Subcomponent 1: Interparental positive interactions elicit child positive emotions

The literature on positive interpersonal processes in adult relationships demonstrates that when adults engage in interactions such as gratitude, capitalization, and shared laughter, these interactions tend to be imbued with positive emotions. For instance, research on capitalization suggests that when individuals share positive events with important people in their lives, doing so can increase the positive emotions associated with that event beyond the individual’s own, initial personal experience of the event (Gable et al., 2006; Reis et al., 2010). Research has documented similar, positive emotion boosts associated with other positive interpersonal interactions, such as gratitude interactions (e.g., Algoe et al., 2016).

What does it mean for children to be present or nearby when parents engage in interactions that are imbued with positive emotions? Families are tightly interwoven systems (Cox & Paley, 2003), meaning that what happens between parents is also likely to impact their children. We know this from extensive research on family systems (Cox & Paley, 2003) and interparental conflict (van Eldik et al., 2020). Moreover, extensive research on affective contagion has revealed that emotions often spread within and across interpersonal interactions (Hatfield et al., 1993; Waters et al., 2017; West et al., 2017). Affective contagion is particularly relevant during familial interactions, when shared affective states can influence the development of healthy self-regulation in children (Harrist & Waugh, 2002; Waters et al., 2017). Indeed, an accumulating body of research demonstrates that affect does indeed spread within families, including from parents to children. For instance, Waters et al. (2014) demonstrated that when mothers were randomly assigned to experience stress while being separated from their infants, on being reunited, their infants tended to mirror the mothers’ physiological experiences. Other studies have similarly demonstrated that when parents tend to experience stress, anxiety, or positive affect, their children will tend to mirror the affective states and the associated psychophysiology of their parents (Kidby et al., 2023; Nook et al., 2023; Smith et al., 2022; Waters et al., 2017, 2020). This evidence provides firm foundation for one of the core predictions of IPST and one of the three subcomponents of spillover: When parents engage in positive interactions, their children will experience positive emotions as a result.

### Spillover Subcomponent 2: Interparental positive interactions beneficially alter children’s perceptions of their parents

Children derive important information about their parents by viewing how they interact with each other. For instance, prior research on interparental conflict indicates that interparental interactions signal important information to children, such as the degree to which the parental relationship is insecure or whether the stability of the family is threatened (Cummings & Davies, 2011; Davies et al., 2016; Davies & Martin, 2014). Even though the child is not directly involved in the interparental conflict, this type of signaling is a critical reason why interparental conflict threatens emotional security and, therefore, children’s well-being and development (Davies et al., 2016; Davies & Martin, 2014).

Conflict, of course, is not the only type of social interaction that conveys information to onlookers. Positive interpersonal interactions also signal important information to individuals who witness them. Algoe et al. (2020), for example, found that when two people engage in gratitude interactions, it changes the way third-party witnesses (observers) view and behave toward both people.^4^ Specifically, third-party witnesses of gratitude interactions have more positive perceptions of both members of the interaction, viewing expressers of gratitude as more responsive and targets of gratitude as better people. Notably, however, this research was conducted with adults who witnessed interactions between individuals with whom they had no prior relationship. Thus, although the research by Algoe et al. (2020) is foundational in documenting that positive interactions signal key information to onlookers, no prior theory or research has considered whether or how positive interparental interactions may signal information to children in a family system, especially information that is distinct from conflictual interactions.

How, exactly, might interparental positive interactions impact children’s views of their parents? Consider interparental expressions of gratitude. The find-remind-and-bind theory of gratitude (Algoe, 2012) posits that expressions of gratitude alert individuals to the presence of a responsive, reliable partner. In other words, when someone expresses gratitude, it makes us aware that the individual who expressed the gratitude is worth spending time and “binding” with. Algoe et al. (2020) demonstrated this social binding function extended even to third-party witnesses of gratitude interactions and that witnesses viewed both individuals in the interaction more positively after viewing the interaction. Applying this work to the family context, IPST suggests this social binding function of gratitude may also apply to children who witness their parents engaging in gratitude interactions. For example, when Jamal witnesses his parents expressing gratitude to each other, he should come to view his parents as more likely to be responsive to his own needs.^5^ This, in turn, should increase his willingness and desire to bond with his parents, bringing the entire family closer together.

Other interparental positive interactions should have similarly positive implications for how children view their parents. For instance, enthusiasm and validation lie at the heart of effective capitalization interactions (Reis et al., 2010). If Jamal witnesses his parents enthusiastically sharing and responding to each other’s good news, this should signal his parents’ trustworthiness (i.e., they can be trusted with prized feelings) and responsive encouragement (i.e., they respond enthusiastically to accomplishments). Thus, Jamal should come to view his parents as more trustworthy and available for support in the future, and a similar process should occur for other positive interparental interactions, such as shared amusement and expressions of admiration.

It is important to note the connection here between IPST and attachment theory. Within attachment theory, beyond providing comfort, safety, and security (i.e., a safe haven; N. L. Collins & Feeney, 2000), attachment figures can also provide a secure base that encourages individual exploration, growth, and goal strivings (Feeney, 2004; Feeney & Thrush, 2010). Specifically, individuals with primary attachment figures who are (a) available (responsive, attentive, and open), (b) encouraging (supportive and enthusiastic), and (c) nonintrusive (not interfering) will feel supported in exploring the world, pursuing their goals, and developing key competencies (Bowlby, 1988; Feeney & Thrush, 2010). According to IPST, by enhancing the child’s perceptions that the parent is responsive and encouraging in a nonintrusive manner, interparental positive interactions contribute to a secure base that supports the child’s ability to engage in exploration, growth, and goal strivings outside of the family unit. Thus, whereas interparental conflict threatens child security and safety, interparental positive interactions enhance the child’s positive perception of parents that fortify a secure base for the child.

### Spillover Subcomponent 3: Children learn how to engage in positive behaviors by watching their parents

Research on social learning demonstrates that children have a natural tendency to observe the behavior of people in their social milieu and, in doing so, acquire key information about behavior (Gruber et al., 2022). Indeed, using strategies such as imitation and emulation, children can obtain information and behavior more efficiently than if they had discovered such information on their own (Csibra & Gergely, 2009; Gruber et al., 2022; Nielsen, 2006).

Scholars have emphasized the importance of affect in the process of social learning. Theory and research on affective social learning suggests that children observe the affect associated with objects and behaviors in the social environment as they choose which behaviors to imitate or emulate (Clément & Dukes, 2017; Dukes & Clément, 2019; Gruber et al., 2022). That is, children can observe specific behaviors and notice the affective “results” that occur in response to those behaviors: Some behaviors tend to trigger joy, laughter, and warmth, whereas others tend to trigger sadness or anger. Research has demonstrated that even young children will alter their behavior on the basis of observing the affective responses of adults in the social environment (Egyed et al., 2013; Reschke et al., 2020).

IPST proposes that the third subcomponent of interparental positivity spillover is social learning. Children are natural learners who pay attention to the affect associated with behavior in their social environments (Clément & Dukes, 2017; Dukes & Clément, 2019; Reschke et al., 2020). By being embedded in a family in which it is normative to initiate and contribute to positive interactions, IPST suggests that children will naturally learn to engage in these behaviors within their own lives (e.g., in their peer relationships, with other adults, and in their relationships with their parents or siblings). This is especially true because interparental positive interactions are imbued with positive emotions: Through affective observation, children will learn that positive social interactions such as sharing positive events, expressions of gratitude, and shared humor tend to produce positive affective results, and children will therefore be more likely to imitate and emulate these behaviors.

## Some Assumptions of the Three Subcomponents of Spillover

**Fig. 1.**
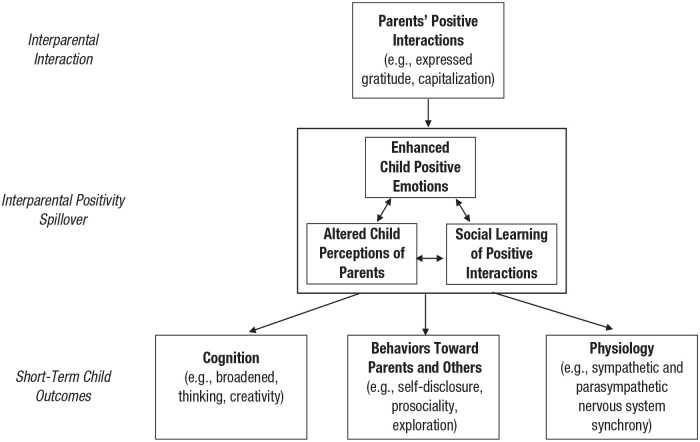
Short-term outcomes of a single positive interparental interaction. “Short term” refers to the outcome of one interaction (e.g., how one positive interparental interaction influences a child’s outcomes on a particular day).

**Fig. 2.**
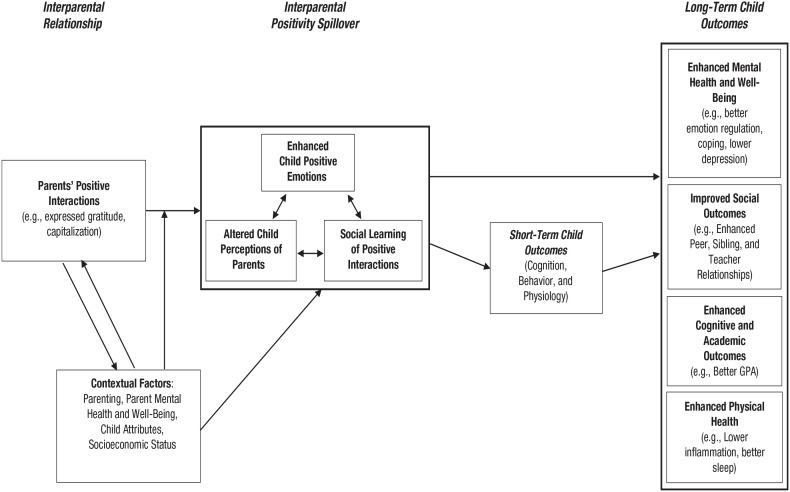
Long-term child outcomes of frequent positive interparental interactions. “Long term” refers to how frequent positive interparental interactions lead to positive child outcomes across months or years.

Now that the three subcomponents of interparental positivity spillover have been outlined, a few notes about assumptions are warranted. Although IPST suggests that each of these subcomponents is a distinct facet of interparental positivity spillover, it is also important to recognize that each of the subcomponents is also likely interrelated with the other subcomponents. Consider children’s perceptions of their parents: Prior research suggests that perceptions and judgments of other people tend to be influenced by transitory mood states, including by positive mood (Forgas, 1995; Forgas & Bower, 1987; Forgas et al., 1994; Goldring & Bolger, 2022). To the extent that children experience positive emotions from witnessing interparental positivity, this also likely (beneficially) influences their perceptions of their parents. Thus, these two subcomponents of interparental positivity spillover, although distinct, may influence each other (as illustrated by the double-headed arrows in Figs. 1 and 2). Likewise, some researchers regard affective contagion itself a form of social learning (Gruber et al., 2022). That is, when children experience contagious positive emotions because of interparental positive interactions, it should spur on children’s imitation and emulation of these behaviors because these behaviors are directly associated with the child’s own experience of positive emotions. Thus, these two subcomponents of interparental positivity spillover may also be intertwined. Finally, the child’s perceptions of their parents may be linked with their likelihood of imitating or mimicking their parents’ social behavior. In sum, although IPST posits that all three subcomponents of spillover are distinct, they are also, to an extent, intertwined and mutually reinforcing.

IPST also suggests that the extent to which interparental positive interactions spill over into child experiences depends on the quality of the interparental interaction. That is, although positive interpersonal interactions such as gratitude tend to be infused with positive emotions and tend to generate beneficial outcomes (Algoe, 2019; Algoe et al., 2013; Gordon et al., 2012), not all positive interactions are created equal. For instance, Algoe et al. (2016) demonstrated that the outcome of gratitude expressions in adult relationships depends on the extent to which the expresser engages in other-focused praising behavior. In capitalization interactions, the outcome of the interactions depends on the extent to which the person responding to the positive event is active and constructive, or enthusiastic and behaviorally engaged (Gable et al., 2006; Peters et al., 2018; Reis et al., 2010). Keeping this research in mind, IPST suggests the extent to which spillover will occur (including all three subcomponents of spillover) depends on the quality of the interaction between the parents (i.e., the degree to which the interaction generates authentic positive emotions and enhanced feelings of connection for the parents).

Related to the above point, prior theory and research also suggest that interparental positive interactions may be especially likely to spill over when they involve both members being actively engaged in the interaction. For instance, theoretical models such as the interpersonal process model of intimacy (Laurenceau et al., 1998; Reis & Shaver, 1988) and positivity resonance theory (Fredrickson, 2016) emphasize that emotionally laden interactions in close relationships are most beneficial when they are characterized by mutual responsiveness and caring, involving both members of the interaction. Using the example of a capitalization interaction, this suggests that if one parent enthusiastically discloses a positive life event to their partner, but their partner fails to respond actively to this enthusiasm, sharing the positive life event is unlikely to generate affective or relational benefits for the individual or the dyad. Research on capitalization, in fact, reveals that even if one individual enthusiastically discloses a positive life event, this disclosure generates positive emotions and relational benefits only if their partner responds in an active and constructive manner (for a review, see Peters et al., 2018). IPST suggests that interparental positive interactions will generate a greater degree of spillover when both parents take an active, constructive role in the interaction compared with when only one parent drives the interaction.^6^

IPST also draws on self-determination theory to suggest that interparental positive interactions will most likely spill over into child outcomes when they are authentic. Self-determination theory proposes that behavior in relationships tends to be beneficial to the extent that it is autonomously motivated and authentically endorsed (Ryan & Deci, 2017). Indeed, extensive research demonstrates that relationship behaviors and self-expressions that are more autonomously motivated, self-endorsed, and authentic are associated with better individual and relational well-being (Al-Khouja et al., 2022; Don & Hammond, 2017; Knee et al., 2013). Although prior research has yet to examine authenticity in this context, some interparental positive interactions are likely more authentic than others. For instance, if Mike’s father feels forced and pressured to express appreciation for Mike’s mother because the extended family is present (e.g., at a holiday gift exchange), this interparental positive interaction would be low in authenticity. If Jamal’s father, on the other hand, genuinely expresses his admiration for Jamal’s mother after she accomplishes a challenging goal at work, this would be a positive interaction higher in authenticity.^7^ Consistent with self-determination theory, IPST suggests that interparental positive interactions will be more likely to beneficially spill over into child outcomes when they are higher in authenticity, meaning that Jamal would be more likely than Mike to experience benefits from witnessing the interparental interactions mentioned above.

Finally, IPST acknowledges that not all seemingly “positive” interparental interactions will beneficially spill over into children. For instance, some positive emotions—such as schadenfreude and hubristic pride—have a dark side (Brown & Fredrickson, 2021; Cikara, 2015; Gruber, 2011). Consider the emotion of schadenfreude, in which people experience pleasure at another person or out-group’s pain (Cikara, 2015; Leach et al., 2003; Smith et al., 2009). Although schadenfreude may feel subjectively good for the individual, prior research demonstrates that this emotion can have negative consequences, such as in motivating violence against derogated out-group members (Cikara, 2015). Even if children view their parents engaging in the shared pleasure of reveling at another person’s misfortune, this type of positive interaction is unlikely to beneficially spill over into children’s experiences, primarily because the interparental interaction is not characterized by warmth and mutual care. Drawing from positive resonance theory (Fredrickson, 2016), which posits that moments of shared positive emotions characterized by care and love are particularly critical to mental and physical health, IPST posits that interparental positive interactions will beneficially spill over into child experiences to the extent that they are characterized by warmth and mutual care. Without these ingredients, positive interparental interactions are less likely to bring parents affectively and relationally closer together and, therefore, unlikely to result in spillover that benefits children.^8^

## The “How”: Understanding How Interparental Positive Interactions Influence Beneficial Child Outcomes via Spillover

A critical assumption of IPST is that interparental positive interactions contribute beneficially to children’s well-being and development. According to IPST, this occurs in the short term because of individual interparental interactions and across the course of time because of frequent and accumulated interparental positive interactions. First, we explicate how this occurs as a result of individual interactions: IPST posits that, by spilling over into child positive emotions, beneficially altered perceptions of their parents, and social learning of positive behaviors, individual interparental interactions beneficially influence their child in that moment. This process includes their child’s cognition (Prediction 2a), their child’s behaviors toward other people (including their parents; Prediction 2b), and their child’s physiology (Prediction 2c). These ideas are depicted in Figure 1. Notably, although IPST predicts that each subcomponent of spillover contributes in some way to children’s beneficial outcomes in the short term, prior research suggests that some of the subcomponents may be especially relevant to particular outcomes (e.g., children’s positive emotions and perceptions of their parents may be particularly relevant to their cognitions). In our review below, we detail which subcomponents of spillover are, in theory, most likely to contribute to each short-term outcome.

### Interparental positivity and children’s short-term cognitive outcomes

With respect to cognition, prior research and theory suggest that interparental positive interactions should have an influence on cognition because of the way they beneficially contribute to (a) positive emotions and (b) perceptions of the parents. With respect to positive emotions, the broaden-and-build theory of positive emotions (Fredrickson, 2001, 2013) suggests that whereas negative emotions tend to narrow one’s focus onto specific problems or threats, positive emotions broaden the scope of an individual’s thought-action repertoire, opening them to a wider range of cognitive and behavioral possibilities. Extensive research among adults has confirmed that positive emotions broaden the scope of attention and contribute to more flexible, creative thinking (for a review, see Fredrickson, 2013), although notable boundary conditions on this effect have been documented (Harmon-Jones et al., 2013). Research examining positive emotions among children similarly suggests that positive emotions contribute beneficially to the cognition of children (Stifter et al., 2020). For instance, studies have experimentally manipulated positive emotions among children and (consistent with the broaden-and-build-theory) demonstrated that positive emotions contribute to better creativity, problem-solving, attention to detail, and simultaneous thinking (Blau & Klein, 2010; Greene & Noice, 1988; Rader & Hughes, 2005). This prior theoretical and empirical work based on the broaden-and-build theory illustrating the cognitive outcomes of positive emotional states provides a firm foundation for IPST’s prediction that interparental positive interactions will promote enhanced short-term child cognitive outcomes via the mechanism of increased child positive emotions. Critically, a significant body of evidence suggests that the short-term cognitive changes associated with positive emotions operate independent of negative emotions (Fredrickson, 2013). What this means is that an absence of conflict is unlikely to engender these cognitive benefits because this absence is unlikely to elicit positive emotions in children. As such, according to IPST, interparental positive interactions play a unique role in contributing to creativity, flexible thinking, and problem-solving because these cognitive benefits are particularly relevant to positive emotions, including among children (Fredrickson, 2013; Rader & Hughes, 2005; Stifter et al., 2020).^9^

IPST also posits that interparental positive interactions contribute beneficially to cognition via the way they influence children’s perceptions of the parents. Specifically, because interparental positive interactions theoretically contribute to enhanced perceptions of parent availability and responsiveness, they will enhance the extent to which children perceive that they have a secure base from which to engage in exploration, challenges, and creativity. Prior research on secure-base priming demonstrates that when people are experimentally reminded of key attachment figures who are available during times of exploration and challenge, it enhances creative problem-solving (Mikulincer et al., 2011) and reduces emotional reactivity in response to frustration during a challenging task (Karreman et al., 2019). Moreover, adult relationships characterized by greater secure-base support (including availability, responsiveness, and encouragement) predict greater exploratory behavior for the adults in these relationships (Feeney & Thrush, 2010). All of this suggests that, after an interparental positive interaction—which theoretically enhances perceptions of the parents—children will be more willing to explore novel, challenging, and creative cognitive tasks.

### Interparental positivity and children’s short-term social outcomes

In addition to these cognitive outcomes, IPST predicts that interparental positive interactions will have numerous beneficial short-term implications for children’s social behavior. Prior research provides reason to suggest all three subcomponents of spillover will mediate the influence of interparental positive interactions on short-term social behaviors. With respect to positive emotions (the first subcomponent of spillover), researchers in the area of prosocial development have argued that parents’ positive emotional expressivity is a key component of children’s emotional socialization that contributes to their social behavior (Eisenberg et al., 2013; Q. Zhou et al., 2002). In this area, expressiveness refers to the parent’s tendency to display positive emotions, both verbally and nonverbally, including in situations that do not directly involve the child. Prior research has documented that when parents display higher levels of positive emotional expressivity, children will tend to engage in more positive social behaviors (Eisenberg et al., 2003; Michalik et al., 2007; Q. Zhou et al., 2002). For instance, in a study by Q. Zhou et al. (2002), when parents demonstrated greater levels of positive emotional facial expressivity in response to a set of emotion-evoking pictures (as rated by independent observers), their children were more likely to display empathy and prosocial behavior. Other research documents that when children experience greater positive emotions, they are more likely engage in prosocial behavior and display social competence (Dougherty, 2006; Isley et al., 1999; Lennon & Eisenberg, 1987). For multiple reasons, then, interparental positive interactions likely foster better social behaviors for children: Instances of interparental shared amusement, gratitude, and capitalization (among other interparental positive interactions) typically involve a high degree of positive emotional expressivity and likely stimulate positive emotions for children. The existing research suggests that this should promote the child’s better social behavior in the ensuing moments after the interaction, including with their parents, siblings, teachers, and peers.

IPST posits that the second subcomponent of positivity spillover—children’s (positive) perceptions of their parents—also plays an important role in their social behavior. According to attachment theory, mental representations of attachment figures guide interpersonal thoughts, feelings, and behaviors (Bowlby, 1988). That is, on the basis of experiences with key attachment figures, children develop perceptions and representations of attachment that guide their behavior in other, attachment-relevant situations. Positive interparental interactions are typically infused with warmth, affection, and positivity; IPST suggests that these interactions typically enhance the child’s perceptions of their parents, as well as their feelings of attachment security in that moment, beneficially altering that child’s social behavior.

Some existing research provides support for the ideas that short-term boosts to attachment security alter social behavior in beneficial ways. Indeed, just as priming attachment security benefits cognitive processes, it likewise benefits socially relevant outcomes. Research on secure-base priming suggests that subliminal or supraliminal reminders of available and encouraging attachment figures have numerous beneficial consequences for social behavior and outcomes, such as in promoting self-transcendent values (Mikulincer et al., 2003), reducing negative reactions to out-groups (Mikulincer & Shaver, 2001), and enhancing positive expectations of relationships (Carnelley & Rowe, 2007). Among studies that examine feelings of attachment security and social outcomes among children, much of the research is longitudinal (e.g., Boldt et al., 2020), meaning little work has specifically examined how short-term (beneficial) changes in child perceptions of parents may alter children’s social short-term social outcomes. Among the existing cross-sectional studies, parent–child attachment security is associated beneficially with social outcomes, such as lower loneliness, better social adjustment, and better social competence (Contreras et al., 2000; Kerns et al., 2006; Kerns & Stevens, 1996). This prior research supports IPST’s proposition that by beneficially altering children’s perceptions of their parents (and therefore attachment-related mental representations) interparental positive interactions will contribute to better child social behavior in the short term.

Extensive social-learning research also demonstrates that children will observe and then subsequently imitate their parents’ social behavior (Dukes & Clément, 2019; Gruber et al., 2022). What this means is that, after interparental positive interactions, children should be more likely to engage in the same types of positive behaviors that their parents just engaged in. Therefore, if parents engage in mutual admiration, shared amusement, or expressions of gratitude, children should be more likely to engage in these behaviors after the interaction—both with their parents and with other people, such as their siblings, peers, and teachers. In support of these ideas, some prior research has demonstrated that children of parents who experience or demonstrate more gratitude are also more likely to experience gratitude (Hoy et al., 2013; Hussong et al., 2020, 2021; Rothenberg et al., 2017). Notably, if children do indeed learn from their parents and engage in behaviors such as expressing gratitude or sharing good news in their own social interactions, these behaviors likely contribute to the same relational and affective benefits that they do for adults (i.e., enhanced positive emotions and increased bonding with close others; Algoe et al., 2013). Thus, after interparental positive interactions, and via social learning, children will likely engage in the positive behaviors similar to what their parents’ model, which should enhance child social outcomes.^10^

### Interparental positivity and short-term changes in children’s physiology

Another way in which parents’ positive interactions may influence their children during specific moments is via children’s physiology, such as in their nonconscious bodily responses to social interactions. One way in which this might manifest is in physiological synchrony (Palumbo et al., 2017), or the extent to which parent–child physiological activity is linked or associated. Indeed, as part of a broader array of caregiving behaviors that support healthy child development, physiological synchrony between parents and their children is crucial for healthy child development, including children’s cognitive, social, and emotional growth (Davis et al., 2017; Feldman, 2007, 2012). With respect to short-term outcomes, we focus on parent–child synchrony in autonomic nervous system activity because (a) prior research has documented that the autonomic nervous system shows changes in response to short-term, affect-laden social interactions (Chen et al., 2021); and (b) extensive research has examined physiological synchrony in the autonomic nervous system in parent–child dyads (for a review, see Palumbo et al., 2017).

The sympathetic nervous system (SNS) governs bodily responses during times of threat or stress, whereas the parasympathetic nervous system (PNS) controls bodily functions related to rest, relaxation, and social engagement (Blascovich & Mendes, 2010; Helm et al., 2014; Porges, 2007). Research examining parent–child dyads suggests that children can “catch” and mirror the physiological responses of their parents, as assessed by their PNS and SNS reactivity. In the aforementioned Waters et al. (2017) study, when mothers were induced to experience high-arousal negative emotions, their infants experienced physiological synchrony with their mothers in SNS activity, whereas when mothers were induced to experience low-arousal positive emotions, their infants experienced physiological synchrony with their mothers in PNS activity. Numerous other studies examining parent–child interactions have revealed that children tend to mirror the physiological experience of their parents (Palumbo et al., 2017), but this often depends on the specific social context or other third variables (e.g., level of family risk; Suveg et al., 2016).

Prior research, therefore, has established (a) the importance of physiological synchrony between parents and their children and (b) the existence of physiological synchrony between parents and their children during parent–child interactions. Yet what about parent–child physiological linkage during interparental interactions? To date, research has exclusively examined how children display physiological synchrony with their parents during interparental conflict. For instance, Li et al. (2020) assessed respiratory sinus arrhythmia (a well-established indicator of PNS activity) during a family-conflict discussion and found that adolescent daughters displayed synchrony with their mothers, but only when coparenting conflict in the interparental relationship was low. This suggests that interparental interactions may, in fact, contribute to physiological synchrony between parents and their children (i.e., it is not just parent–child interactions that contribute to synchrony). Building on these results, IPST proposes that positive interparental interactions may also contribute to physiological synchrony between children and their parents, even when the child is not directly involved in these interactions. That is, when parents engage in warm and mutually caring positive interactions, it likely promotes physiological attunement between parents and their children, a pattern that could have beneficial consequences for both the parent–child relationship and numerous other child outcomes, such as the child’s cognitive, social, and emotional growth (Davis et al., 2017; Feldman, 2007).

## IPST and Long-Term Child Outcomes

In addition to the short-term impact of positive interactions during particular moments or days, another primary prediction of IPST is that beneficial outcomes of interparental positive interactions accumulate across time. One useful parallel is to the “build” portion of the broaden-and-build theory of positive emotions (Fredrickson, 2001, 2013). This theory posits that although any single instance of positive emotion may be fleeting or transitory, over time the frequent recurrence of positive emotions tends to accumulate to build and strengthen enduring and consequential resources, including mental, physical, and social resources. An extensive amount of evidence supports this proposition (for a review, see Fredrickson, 2013). IPST adopts a similar stance with respect to the influence of interparental positive interactions on children: When parents frequently engage in positive interpersonal interactions, this represents a recurrent infusion of positivity into the family system, which can accumulate in beneficial ways beyond short-term moments. Thus, IPST posits that parental relationships characterized by frequent positive interactions should build children’s resources over time and in doing so enhance children’s well-being and healthy development (Prediction 3). These ideas are presented in Figure 2. As depicted there, IPST suggests that when parents routinely engage in positive interactions, this will spill over into children’s everyday lives in the form of (a) an increased frequency of positive emotions, (b) enduringly enhanced child perceptions of their parents, and (c) solidified child social learning of these behaviors. Across time, the accumulation of frequent interparental positivity spillover will thereby contribute to enhanced child mental health and well-being (Prediction 3a), social outcomes (e.g., with peers, teachers, and siblings; Prediction 3b), cognitive and academic outcomes (Prediction 3c), and physical health (Prediction 3d). We expand on each of these predictions below, once again noting where each subcomponent of spillover is most likely to contribute to a particular long-term outcome.

### Interparental positivity and children’s mental well-being across time

IPST posits that enhanced mental well-being in children—such as better emotion regulation and lower rates of depression and anxiety—should be one of the central long-term benefits of frequent exposure to interparental positive interactions. According to IPST, this is because interparental positive interactions spill over into (a) children’s positive emotions and (b) their beneficially altered perceptions of their parents.

Why should frequent experiences of positive emotions contribute to children’s enhanced mental well-being across the course of time? Theory and research in the literature on adults have demonstrated how positive emotions enhance coping: By opening individuals to a broader range of thoughts and behaviors, positive emotions enable flexible and adaptive coping, especially when stressors arise (Folkman, 2008; Folkman & Moskowitz, 2000; Ong et al., 2006). Indeed, positive emotions have an extensive and well-established track record of promoting resilience and well-being in ways that are independent of negative emotions (Fredrickson et al., 2003; Gloria & Steinhardt, 2016; Tugade & Fredrickson, 2004). Only a few studies have explicitly tracked children’s positive emotions in everyday life and examined how they contribute to the child’s mental health and well-being across the course of time. Among the studies that do exist, however, evidence suggests that children who frequently experience high levels of positive emotions across time will experience benefits in terms of their mental adjustment (Coffey et al., 2015, 2023; Davis & Suveg, 2014; Dougherty et al., 2010). For instance, Dougherty et al. (2010) examined children’s positive emotionality at age 3 and found that it predicted their tendency to experience lower levels of depressive symptoms at age 10, even accounting for the child’s negative emotionality. Other research from developmental psychology suggests that positive emotions likely have protective benefits for the development of psychopathology, such as depression and anxiety (for a review, see Davis & Suveg, 2014). Taken together, the existing adult and developmental literature on positive emotions and mental health suggests that children of parents who engage in a greater frequency of positive interactions will, across time, experience better mental health and well-being because of the frequency with which they experience positive emotions in everyday life.

In addition to positive emotions, interparental positive interactions should enhance child mental health and well-being across the course of time because of the way they beneficially alter children’s perceptions of their parents. According to IPST, if Jamal’s parents were to frequently engage in positive interactions, this would represent a consistent reminder that his parents are validating, reliable, and encouraging, thereby fortifying the extent to which Jamal’s parents demonstrate their ability to serve as a secure base. This quality of Jamal’s attachment to his parents likely has numerous long-term consequences for his mental well-being. In particular, attachment theory suggests that key attachments serve as an emotional regulatory system for children that enables healthy and effective coping when they are exploring the environment or become distressed (Brumariu, 2015). In support of attachment theory, research demonstrates that the quality of parent-child attachment is associated with children engaging in more effective coping strategies (Gaylord-Harden et al., 2009; Kerns et al., 2007; Psouni & Apetroaia, 2014). Indeed, a meta-analysis of 72 studies demonstrated that the quality of parent–child attachment (including secure-base attachment) contributes to how children regulate their emotions, with more securely attached children utilizing more effective coping strategies (Cooke et al., 2019). Moreover, a meta-analysis of 120 studies examining attachment-security priming among adults similarly concluded that experimentally manipulating feelings of attachment security significantly reduced mental health challenges such as depression, anxiety, and distress (Gillath et al., 2022). This work provides support for the predictions of IPST: Frequent interparental positive interactions will serve as consistent reminders of attachment security, thereby enhancing child emotional regulation, coping, and therefore mental well-being across the course of time.

Given that IPST suggests that both enhanced positive emotions and children’s perceptions of their parents contribute to children’s mental well-being across time, one question that arises is whether one of these subcomponents might be especially likely to promote children’s long-term mental well-being. Because extensive research and theory suggest that both of these subcomponents typically contribute to children’s long-term mental well-being, IPST offers no predictions regarding the relative importance of positive emotions or perceptions of parents in contributing to children’s mental well-being over time. Rather, both of these subcomponents probably play unique and complementary roles in mediating the long-term beneficial effects of positive interparental interactions on children’s mental well-being across time.

### Interparental positivity and children’s social outcomes across time

Another long-term consequence of frequent interparental positive interactions should be improved long-term child social outcomes, including better relationships with peers, siblings, and/or teachers. According to IPST, all three components of interparental positivity spillover—positive emotions, enhanced perceptions of parents, and social learning—contribute to children’s enhanced social well-being in the long-term. With respect to positive emotions, the broaden-and-build theory posits that the accumulation of frequent positive emotions across the course of time enables people to build consequential social resources, and research among adults has demonstrated that positive emotions enable people to build social connections across time (Fredrickson, 2013; Fredrickson et al., 2008; Waugh & Fredrickson, 2006). Only a few studies on development have tracked children’s positive emotions and linked them to social outcomes across the course of time. The studies that do exist, however, provide suggestive evidence for the building function of positive emotions among children. For instance, Volbrecht et al. (2007) demonstrated that positive affect among infants longitudinally predicts their subsequent empathy-related helping behavior. Other studies have demonstrated that positive emotions are associated with social well-being outcomes, such as higher peer social status (Dougherty, 2006). Integrating this theory and research within the body of work on adult affect and development, IPST suggests that the positive emotional spillover that occurs in response to interparental positive interactions allows children to build social resources across the course of time.

In addition to positive emotions, IPST posits that children’s altered perceptions of their parents play a mediating role in their enhanced social outcomes across the course of time. Over time, children’s altered perceptions of their parents should accumulate into stably positive attachment mental representations that enable them to form healthy relationships throughout their everyday life. Moreover, IPST’s prediction that interparental positivity will be especially relevant to fostering secure-base attachment suggests that children of parents frequently engaging in positive interactions will be especially likely to explore new connections and experiences in the social environment (e.g., a willingness to seek out new social connections, a propensity to explore new social groups). Supporting these ideas, research on development has demonstrated that more secure parent–child attachment is associated with better child social outcomes across the course of time. For instance, a meta-analysis of 44 studies demonstrated that more secure parent–child attachment was longitudinally associated with better child peer relationships (Pallini et al., 2014). In addition to peer relationships, given that better parent–child attachment security is associated with children’s social competence (Neppl et al., 2019), children who frequently experience positive perceptions of their parents are also likely to form better relationships with other key people—such as teachers and siblings—in their social environment across time. In sum, the existing literature provides evidence for IPST’s prediction that, when children frequently witness their parents engage in positive interactions, it results in frequent positive perceptions of the parents that accumulate into social well-being outcomes such as better relationships with peers, siblings, and teachers.

IPST additionally suggests social learning of positive interactions also plays a role in promoting positive social outcomes for children across time. IPST posits that one of the short-term consequences of interparental positive interactions is that children will emulate their parents’ positive social behaviors. In addition to having short-term benefits, when children have parents who frequently engage in positive interactions, children (a) are likely to become adept at these behaviors and (b) frequently engage in these behaviors throughout their own lives and social spheres. If Jamal’s parents frequently share in kind-hearted amusement, Jamal himself is likely frequently engage in this same kind-hearted amusement throughout his own life. Over the course of time, this positive behavioral emulation likely provides social benefits for Jamal in the form of enhanced relations with his peers, teachers, siblings, and parents.

With respect to children’s long-term outcomes, IPST suggests that all three subcomponents contribute to children’s long-term well-being. However, IPST claims that beneficially altered perceptions of parents should be the strongest predictor of children’s beneficial long-term social outcomes. This is because of extensive research documenting that children’s secure-attachment representations play a critical role in their ability to form and fortify positive, healthy social bonds (Neppl et al., 2019; Pallini et al., 2014). Although both positive emotions and social learning matter, IPST asserts that one of the most important ways in which interparental positive interactions contribute to long-term social outcomes is by enhancing positive mental representations of parents.

### Interparental positivity and children’s cognitive and academic outcomes across time

In addition to social well-being, IPST posits that, across the course of time, frequent interparental positive interactions accumulate into benefits for children’s cognitive and academic performance. The principal mediators for the long-term benefits of interparental positive interactions on children’s cognition and academic performance are (a) positive emotional spillover and (b) enhanced child perceptions of parents. As outlined above, in the short term, positive emotions momentarily broaden awareness, which paves the way for more flexible, creative thinking, an effect that has been documented in both adult populations and among children. Although research examining child positive emotions and academic outcomes is relatively scarce (Valiente et al., 2012), some prior research has indeed documented the link between greater child positive emotions and better academic outcomes (Coffey, 2020; Hernández et al., 2016; Pekrun et al., 2004). A developmental perspective on the broaden-and-build theory suggests the frequent experience of positive emotions (which occur because of frequent interparental positivity spillover) will result in consistently curious, flexible, and creative thinking in daily life. Over time, this should accumulate (or build) into better academic performance for children, being reflected in outcomes such as their GPA and test scores.

In addition to positive emotions, the attachment-fortifying, beneficially altered perceptions that result from frequent positive interactions should play a critical role in promoting children’s long-term cognitive and academic outcomes. Prior research suggests that attachment security plays a role in children’s academic success. For instance, one study demonstrated that attachment security in toddlerhood could predict academic achievement a decade later (Dindo et al., 2017). Numerous other studies have demonstrated that the quality of parent–child attachment—including secure-base attachment—plays a key role in contributing to children’s cognition and academic achievement over time (for a review, see Bergin & Bergin, 2009). This existing research supports IPST’s prediction that children who frequently witness interparental positive interactions, and thereby have persistent reminders that their parents are validating, encouraging, and responsive, should experience better cognitive and academic outcomes in the ensuing months and years.

With respect to cognitive and academic outcomes, prior research and theory provide good reason to believe that both positive emotions and enhanced perceptions of parents provide benefits. Thus, in this domain, IPST makes no predictions about the relative importance of these two subcomponents in longitudinally predicting cognitive and academic success.

### Interparental positivity and children’s physical health across time

IPST suggests that frequent interparental positive interactions should also accumulate to enhance the physical well-being of children over time. According to IPST, of the three subcomponents of spillover, the primary mediator of interparental positive interactions on child physical health over time is enhanced child positive emotions. This proposition is based on theory and research from affective science that suggests that positive emotions contribute to enhanced physical health across time (Kok et al., 2013; Pressman & Cohen, 2005; Pressman et al., 2019). For instance, because they broaden the scope of attention and behavior, broaden-and-build theory posits that positive emotions promote better physical health across time by engendering feelings of social connectedness, enhancing coping, and altering psychophysiological experiences (Fredrickson, 2001, 2013; Fredrickson et al., 2008; Kok et al., 2013). Indeed, an extensive amount of research demonstrates that positive emotions promote various aspects of physical well-being (Pressman et al., 2019). For instance, people who experience greater positive emotions tend to have lower levels of inflammatory cytokines (Ironson et al., 2018; Stellar et al., 2015), engage more frequently in positive health behaviors (Fredrickson et al., 2021; Nylocks et al., 2019), and recover better when faced with stressors (Folkman, 2008), all of which tend to promote physical health. Over time, IPST predicts that children with parents who frequently engage in positive interactions will receive frequent positive affective boosts that sustain their positive emotions, thereby promoting their physical well-being in the form of lower inflammation, better sleep, and more engagement in health-promoting behaviors.

We note that, although IPST suggests that positive emotions are the primary direct mediator of interparental positive interactions on child physical health across time, this is not the only way in which interparental positive interactions are likely to influence child physical well-being. Many of the other long-term outcomes that result from interparental positive interactions may also contribute to physical well-being. For instance, children’s effective emotional regulation, mental well-being, and social relationships all contribute to their physical wellness (Allen et al., 2015; Keenan-Miller et al., 2007; Trudel-Fitzgerald et al., 2017). To the extent that repeated interparental positive interactions foster these long-term outcomes, they are also likely, over time, to promote children’s physical health.^11^

## The Connection Between Short-Term Outcomes of Interparental Positive Interactions and Long-Term Benefits

Thus far, when discussing the long-term outcomes of interparental positive interactions for children, we have focused on how the three subcomponents of spillover are theoretically likely to contribute directly to beneficial child outcomes across the course of time. In addition to positive emotions, enhanced perceptions of the parents, and social learning longitudinally accumulating and thereby promoting enhanced long-term child outcomes, IPST hypothesizes that individual interparental positive interactions have short-term benefits to cognition, behavior, and physiology; according to IPST, these short-term cognitive and behavioral outcomes also explain why frequent interparental positive interactions, when accumulated across the course of time, promote long-term benefits. For instance, according to IPST one of the principle short-term benefits associated with interparental positive interactions is more positive social behavior. Children of parents who frequently engage in positive interactions ought to regularly engage in more positive and prosocial interactions with their parents, peers, and teachers in the moments and hours after their parents have positive interactions. These short-term benefits serve as an additional mediator through which interparental positive interactions contribute to long-term child benefits, as shown in Figure 2 (Prediction 4: the short-term, beneficial cognitive, behavior, and physiological outcomes following positive interparental interactions should partially mediate the beneficial influence of frequent positive interparental interactions on their children’s long-term outcomes).

## Understanding the Dynamic Interplay Between Positive Interparental Interactions and Conflict in Contributing to Children’s Outcomes

A crucial assumption of IPST is that interparental positive interactions have a beneficial effect on children’s adjustment that is unique and independent of parental conflict. Yet IPST also addresses another important question: How do positive and negative interparental interactions influence each other with respect to children’s outcomes? In the families of Mike and Jamal, interparental conflict was not a major issue. However, in many families, parents have some amount of conflict that exists alongside their frequent positive interactions (e.g., Zemp et al., 2014, 2019). On the basis of research in family, relationship, and affective science, we envision at least three possibilities for the dynamic interplay between negative interparental interactions, positive interparental interactions, and children’s outcomes. First, consistent with the broaden-and-build theory, in which positive emotions produce beneficial outcomes independent of negative emotions (Fredrickson, 2013), one possibility is a *main effect* of positive interparental interactions on children’s outcomes that is independent of the impact of negative interparental interactions. In this case, the association between positive interparental interactions and child outcomes would not be related to the level of interparental conflict (or vice versa).

Some existing research suggests that this main effect pattern is possible. Among studies that have examined how positive emotions contribute to well-being outcomes, some work has shown that positive emotions predict well-being outcomes, independent of negative emotions (Don et al., 2022; Fredrickson, 2013). For instance, in a longitudinal study of couples undergoing the transition to parenthood, Don et al. (2022) found that new parents’ positive emotions beneficially predicted their relationship adjustment, independent of their negative emotions and several other covariates. Likewise, some (but not all) studies of interparental conflict examining parents’ constructive conflict behavior (alongside their hostile or destructive behavior) have found that constructive behaviors during conflict tend to have positive associations with child outcomes, independent of the maladaptive influence of destructive conflict behavior (McCoy et al., 2013; Warmuth et al., 2020). Viewed together, this research provides support for the possibility that interparental positive interactions uniquely and independently contribute to positive child outcomes.

A second possibility involves a buffering hypothesis in which positive interparental interactions may dampen or reduce the deleterious effects of high levels of negative interparental interactions. If, for example, a child’s parents engage in conflicts frequently, frequent gratitude or capitalization interactions between the parents may mitigate the typically negative impact of conflict on the child’s outcomes. Indeed, research demonstrates that positive emotions can buffer the deleterious impact of stressful life circumstances on individuals’ mental health and overall well-being (Algoe & Fredrickson, 2011; Fredrickson et al., 2003; Pressman & Cohen, 2005). Moreover, research on intimate relationships has found that positive relational experiences can buffer the deleterious outcomes often associated with negative relational events (Walsh et al., 2017; Yuan et al., 2010). Research also demonstrates that positive emotions have the capacity to “undo” the physiological effects of negative emotions (Fredrickson et al., 2001; Fredrickson & Levenson, 1998; Yuan et al., 2010), meaning that interparental positive interactions may curtail the maladaptive child physiological outcomes associated with interparental conflict. In addition, although they do not directly examine parents’ positive relational interactions, some studies demonstrate that parents’ constructive conflict behaviors buffer the maladaptive effects of destructive behaviors on child outcomes (e.g., N. Zhou et al., 2021). Considered together, therefore, this evidence suggests positive interparental interactions could temper the negative influence of interparental conflict on children’s outcomes.

A third possibility is a *balance* hypothesis that posits that the key to understanding the interplay between positive and negative interparental interactions in predicting children’s outcomes is the proportion of positive relative to negative interactions (Gottman, 1993; Zemp et al., 2019). According to this perspective, although virtually all parents engage in both positive and negative interactions to some extent, the central driver of children’s outcomes is the proportion of positive to negative interactions (Gottman, 1993). When parents engage in relatively more positive interactions and relatively fewer negative interactions, their children’s outcomes should be better. On the other hand, when parents are not able to balance their negative interactions with more frequent positive ones, this will result in problems for their children.

Empirical evidence from numerous prior studies suggests this balance hypothesis may be accurate. For instance, research examining the health and well-being of intimate couples indicates that couples who have a higher proportion of positive to negative enacted behaviors are at lower risk of divorce and report higher marital quality (Bertoni & Bodenmann, 2010; Gottman & Levenson, 1992). In one of the only existing studies to examine how interparental positive interactions influence children, Zemp et al. (2019) extended this to the family context and found that a higher ratio of positive to negative interparental interactions was associated with better child adjustment. Prior research, therefore, supports the possibility that the balance between parents’ positive and negative interactions contribute to children’s outcomes.

Prior research and theory provide reason to believe that each of these patterns (i.e., the main effect, buffering, and balance patterns) is plausible, which raises the question as to which one IPST anticipates is most likely to occur. Because the current literature suggests that each one is plausible, IPST makes no strong predictions about which pattern is the strongest. Instead, each of these patterns may operate depending on the specific context, time scale, or outcome of interest. For instance, when it comes to the short-term influence of conflict on child perceptions of their parents, a positive interaction immediately after the conflict may at least partially buffer (i.e., moderate) the maladaptive influence of the conflict on the child’s perceptions of their parents. Across the course of time, it may be that families need a good balance of positive to negative interactions for children to experience healthy mental, social, academic, and health outcomes. Because little research has examined how parents’ positive interactions influence children (while also considering the impact of conflict), research is needed to test these possibilities.

## Do Children Need to Be Present for Positive Interparental Interactions to Lead to Benefits?

IPST also addresses whether children must be physically present for positive interparental interactions to be beneficial. Although this remains an empirical question for future research, speculative answers informed by existing theory and research can be offered. Specifically, spillover should occur depending on whether interparental interactions can exert an influence on children’s positive emotions, perceptions of their parents, or social learning of positive interactions. Thus, when it comes to positive emotions, children need not necessarily witness positive interparental interactions for them to spill over into an affective experience. Waters et al. (2017), for instance, showed that when mothers are separated from their children and induced to experience positive affect, this contributes to physiological covariation with their children after being reunited with them. Thus, if Jamal’s parents have a positive interaction in their bedroom and then have dinner with Jamal in the living room, at least one component of spillover is still likely to occur as a result of affect contagion.

With respect to children’s perceptions of their parents and social learning of behaviors, however, we believe that it is more important that children be present and witness interparental positive interactions. In Algoe et al.’s (2020) research on witnessing, which found that positive interactions alter perceptions of the individuals who are engaging in the interactions, all participants directly viewed the positive interactions. Additionally, fundamental to the idea of social learning of behavior is the notion that children must view behaviors (including the affect associated with the behaviors) to eventually enact them.

In sum, do interparental positive interactions need to be witnessed by children to yield benefits? We believe the answer is that they should have greater benefits if children witness these interactions, but they may still have some benefits if children do not witness them. This is because one of the three subcomponents of spillover—positive emotions—can still impact children and contribute to their positive outcomes, even if they do not necessarily witness a positive interparental interaction.

## Do Different Types of Interparental Positive Interactions Contribute Differently to Child Outcomes?

Jamal may witness his parents engage in many different types of positive interactions. Throughout this article, we have focused on some of the most well-studied examples, including expressions of gratitude, capitalization interactions, and shared laughter. However, other examples exist, including expressions of admiration, shared awe, and infectious enthusiasm. Thus, another important question arises: When shared between parents, do each of these positive interactions influence children in a different way?

The primary goal of IPST is to describe the general spillover function of positive interparental interactions as a broad class of behaviors. That is, IPST suggests that positive interparental interactions—such as gratitude, capitalization, shared laughter, and expressions of admiration—although distinct from each other, share the function of spilling over into (a) child positive emotions, (b) beneficially altered perceptions of the parents, and (c) social learning of positive social behaviors. In making this assumption, IPST borrows from the broaden-and-build theory (Fredrickson, 2001, 2013), which posits that positive emotions such as joy, interest, love, and contentment, although phenomenologically distinct, collectively share the ability to broaden thought-action repertoires and build resources. Like the broaden-and-build theory, IPST posits that positive interparental interactions beneficially contribute to child outcomes (via the three subcomponents of spillover), regardless of the specific type of interaction.

Although IPST posits that interparental positive interactions share a broad positivity spillover function, it also acknowledges that each type of positive interaction is unique and may result in a different “flavor” of short-term spillover. For instance, some of the emotional content that is characteristically embedded in a gratitude (shared gratitude) versus capitalization (shared joy) interaction is unique.^12^ Because of this, although IPST predicts children will experience elevated positive emotions after both types of positive interactions, they may experience a different mix of discrete positive emotions after a gratitude versus capitalization interaction. Although Jamal is likely to experience elevated feelings of joy and love after both types of interactions, he may be especially likely to feel grateful after witnessing the gratitude interaction, and especially likely to feel excitement after the capitalization interaction. Likewise, although IPST predicts children will typically experience beneficially altered perceptions of their parents, the specific ways in which their perceptions are altered may be different after each type of interaction. After witnessing a gratitude interaction, Jamal might perceive his father as especially kind, whereas after a capitalization interaction, he might perceive his father as especially enthusiastic. The same is true for how interparental positive interactions will influence child behavior. Algoe et al. (2020) demonstrated that, compared with a general positive-expression control condition, third-party witnesses of a gratitude expression were more likely to want to help, self-disclose to, and affiliate with the gratitude expresser. This suggests that, after his parents engage in a gratitude interaction, Jamal may be especially likely to help, self-disclose to, and affiliate with his parents. These short-term flavors of spillover may be of interest from a basic research perspective.

With the exception of research by Algoe et al. (2020), very little work has examined how positive interpersonal interactions influence third parties (see also Vaish & Savell, 2022). When examining hypotheses related to IPST, we encourage researchers to include assessments of children’s (a) discrete positive emotions, (b) perceptions of multiple characteristics of their parents, and (c) numerous theoretically relevant behaviors. This would allow for an examination of the general spillover function of interparental positive interactions, as well as an exploration of the unique influence that one type of interaction may have on specific child positive emotions, perceptions of the parents, and behaviors.

Notably, from the perspective of IPST, which details the long-term benefits to the child from cumulative instances of interparental positivity, we expect that, relative to parents who express little positivity together, parents who express one type of positivity (e.g., shared laughter) also likely express another (e.g., awe). This means, in families in which parents frequently engage in positive interaction, a child likely—across time—gets a suite of general benefits that are captured by our broad predictions about the long-term effects we detail in Figure 2. As such, although the benefits of an individual interaction may have a unique flavor, across the course of time children are likely to experience general enhancements in positive emotions, perceptions of their parents, and learning of positive behaviors in response to their parents’ frequent positive interactions.

## Interparental Positivity in an Open Developmental System

Interparental interactions do not occur in a vacuum. That is, when parents engage in behaviors such as expressions of gratitude, they always occur in the context of other key parental, child, and familial characteristics, such as each parent’s mental health status and the developmental stage of their child. In the EST literature, researchers have suggested that interparental conflict occurs in the context of an “open developmental system,” whereby interparental conflict both shapes, and is shaped by, other familial contextual factors, such as parenting behaviors, parental mental health, and children’s attributes (Davies & Martin, 2014). Although EST posits that interparental conflict directly contributes to children’s emotional security, it also suggests that conflict influences other aspects of the family system, which in turn have implications for children’s emotional security and adjustment. For instance, interparental conflict may negatively affect parenting behaviors and parents’ mental health, factors that also have a negative bearing on emotional security (Davies & Martin, 2014). Thus, in addition to its direct influence, interparental conflict may also have an indirect influence on children’s emotional security and other outcomes via its deleterious impact on other aspects of the developmental system (e.g., parenting behavior, parent mental health). Likewise, just as parents’ mental health (e.g., depression) may act as a mediator between interparental conflict and children’s emotional security, it may also moderate the effect of interparental conflict on children’s emotional security. Some research, for instance, indicates that interparental conflict is especially harmful for children of parents with depression (Kouros et al., 2008). These examples suggest that interparental interactions need to be considered within the broader familial context in which they occur.

IPST similarly acknowledges that interparental positive interactions do not occur in a vacuum. Interparental capitalization interactions, expressions of gratitude, shared laughter, and other positive interactions always occur within a broader familial context, which includes factors such as parenting behaviors, parental psychopathology, and child attributes and characteristics (e.g., age, gender, temperament). Just as EST posits a transactional, dynamic set of relations between (a) interparental conflict, (b) contextual characteristics such as parenting behaviors and psychopathology, and (c) the child’s emotional security and adjustment (Davies & Martin, 2014), IPST also posits a transactional, dynamic set of relations between (a) interparental positive interactions, (b) contextual familial characteristics, and (c) a child’s needs and adjustment (for an illustration, see Fig. 2).^13^

Consider an important contributor to child well-being and adjustment: parental warmth. When parents are more responsive, caring, and sensitive to their child’s needs, these actions have a host of beneficial outcomes for children, both during childhood and across the course of time (e.g., Moran et al., 2018; Padilla-Walker et al., 2016; Q. Zhou et al., 2002). Thus, any aspect of the family system that contributes to greater parental warmth should indirectly promote child well-being. Indeed, IPST posits that interparental positive interactions not only directly contribute to child outcomes via spillover, but also indirectly contribute to child well-being via other key contributors to child well-being, such as more warm and effective parenting (Prediction 5: interparental positive interactions indirectly influence child well-being via contextual factors, such as parenting behavior and parent mental health). Extending the example of Jamal, if Jamal’s parents frequently engage in expressions of gratitude or admiration for each other, IPST predicts these positive interactions will tend to generate warmth and positivity, ultimately promoting their warm and responsive parenting of Jamal.

In addition, contextual factors, such as aspects of the environment, characteristics of Jamal’s parents, or attributes of Jamal himself, may moderate the extent to which interparental positive interactions contribute to spillover and, subsequently, to Jamal’s healthy adjustment (Prediction 6: contextual factors moderate the influence of interparental positive interactions on child well-being).^14^ This portion of the model, therefore, is a framework for understanding risk and resilience, including those children and families who are most (versus least) likely to benefit from interparental positive interactions. For example, research addressing positive interpersonal processes indicates that people who score high in social approach motivation tend to seek out and respond to positive social events and interactions particularly strongly and derive greater affective and social benefits from these interactions (Don et al., 2022; Elliot et al., 2006; Gable, 2006). As such, one child attribute that might be relevant to Jamal’s response to his parents’ positive interaction is his social approach motivation. If Jamal is strongly motivated to seek out and respond to positive social experiences, he may benefit more when his parents engage in positive interactions. Additionally, biological and genetic factors may influence how children respond to parents’ positive relationship interactions. Research on vantage sensitivity has found that genetic factors predispose some people to respond more strongly after exposure to positive experiences in the environment (e.g., Pluess, 2017). Likewise, research suggests that biological predispositions can influence how people perceive and experience a positive interpersonal process. For instance, Algoe et al. (2017) demonstrated that people with higher levels of oxytocin tend toward enhanced perceptions of the partner and love during gratitude interactions (see also Algoe & Way, 2014; Chang et al., 2022; Isgett et al., 2016, 2017). These findings suggest some children may be genetically or biologically predisposed to experience especially strong benefits in response to interparental positive interactions.

Another important question is what makes children resilient to low levels of interparental positivity in the family system. Although IPST predicts that low levels of interparental positivity are a risk factor for poor child outcomes, research on developmental resilience suggests numerous factors that may mitigate the impact of low interparental positivity, such as high child self-efficacy, optimism, or the presence of another supportive adult in the child’s life (Masten, 2019). We note here, too, that genetics and biological predispositions likely play a key role in resilience. Viewed more broadly, these examples illustrate that not all children are likely to respond to interparental positivity in the same way. Although IPST predicts that interparental positive interactions will generally affect children as outlined in Predictions 2 and 3, because children are nested in complex family systems, their responses to these interactions are likely to vary depending on a number of biological, genetic, and contextual factors.

This portion of the model also speaks to another important topic—the factors that contribute to interparental positive interactions. Much of the prior research examining positive interpersonal processes has focused on their nature and function (e.g., Algoe et al., 2013; Gable et al., 2006; Kurtz & Algoe, 2015), as opposed to the factors that contribute to whether or not people engage in these processes (for exceptions focusing on the role of the oxytocin system in contributing to gratitude expressions, however, see Algoe et al., 2017; Chang et al., 2022). An exhaustive overview of the factors that may contribute to greater engagement in positive interpersonal processes is beyond the scope of IPST. Despite this, we draw on the existing literature to propose a few factors that should be likely to affect which parents are more versus less likely to engage in positive interpersonal processes.

According to IPST, the extent to which parents engage in positive interactions depends on a host of individual-difference (e.g., biological predispositions and personality), relationship-specific (feelings of satisfaction and commitment), family and child (coparenting quality and child behavior problems), and socioenvironmental (culture, socioeconomic status, and neighborhood safety) variables. We briefly provide one example that is particularly relevant to IPST: parents’ coparenting quality.

Coparenting refers to the extent to which parents successfully navigate the shared demands of parenting. It involves behaviors such as supporting each other in the parenting role, navigating differences in beliefs about how to raise the child, and splitting the division of labor in a way that feels fair (Feinberg, 2002; Feinberg et al., 2012). Coparenting has a well-established influence on key outcomes such as parental negativity, relationship satisfaction, mental health, and child maladjustment (e.g., Don et al., 2013; Feinberg et al., 2007; Solmeyer & Feinberg, 2011), but research has yet to examine how it contributes to interparental positive interactions. Among numerous child and family variables that may impact the extent to which parents engage in positive interactions, IPST posits that parents who are successful in coparenting should also be more likely to express gratitude, enthusiastically share positive life events, express admiration, and engage in other types of positive interactions.^15^

## The Role of Culture

One other important consideration is the role of culture in influencing the way in which interparental positive interactions might shape the well-being and development of children. Consider interparental gratitude interactions: Theory suggests that the expression of gratitude serves a social binding function, alerting individuals to good, responsive relationship partners, and helping bind them together (Algoe, 2012). Although this social binding function of gratitude is theorized to occur across cultures, the way in which gratitude is expressed and perceived may be different depending on the cultural context (Chang & Algoe, 2020). Chang and Algoe (2020), for instance, showed that it is more common for people in the United States to demonstrate gratitude with bodily contact (e.g., a hug or a handshake), whereas people in Taiwan are more likely to demonstrate gratitude by stating their desire for self-improvement. This indicates that gratitude is expressed and displayed in culturally specific ways in the United States and Taiwan.

This difference in the expression (but not the function) of positive interpersonal interactions in different cultures is an important consideration for IPST. We suggest that (a) positive interparental interactions occur in many different cultures but that (b) these interactions may have a different form in different cultures. For instance, the specific way that parents engage in shared amusement, express gratitude, or share good news in their relationship may look different in the United States, Taiwan, or Botswana. Regardless of how positive interparental interactions are expressed in a particular culture, the predictions of IPST should remain consistent across cultures in that (a) short-term, interparental positive interactions should have beneficial consequences for children; and (b) across time, consistent and frequent interparental positive interactions should accumulate into the beneficial well-being and development of children.

Prior research has examined other cultural considerations relevant to IPST. Research on ideal affect, for example, reveals that individuals from East Asian cultures tend to value and display high-arousal positive emotions more than low-arousal positive emotions (Tsai & Clobert, 2019). This suggests that parents’ affect valuation (i.e., the emotions that they value and would like to feel) may be an important individual-difference variable relevant to interparental positivity spillover in that parents who value high-arousal positive emotions may be more likely to (a) engage in interparental positive interactions and (b) have children who benefit from them. In addition, although we have primarily focused on comparisons between East Asian and Western cultures when discussing culture thus far, research has document numerous psychological processes in other cultural contexts that may influence how interparental positivity spillover unfolds (e.g., in Mediterranean and Latino cultures; Senft et al., 2021; Uskul et al., 2023). Importantly, most of the research that supports the principles and predictions of IPST has been conducted with samples that are not representative of the global population (i.e., Western, educated, industrialized, rich, and democratic, or WEIRD, couples and families; Henrich et al., 2010). Thus, IPST must remain open to revision as we learn more about positive interparental interactions in non-WEIRD families.

## Implications for Intervention

Translating theory and empirical research into applied interventions with the potential to help children and families remains a top priority for research in family science. For instance, journals such as the *Journal of Family Psychology* encourage authors to advance work that has the potential to translate basic research into effective interventions (Connell, 2022). Moreover, translational research is a high priority for funding agencies such as the National Institutes of Health (F. S. Collins, 2011). Thus, it is important to consider how IPST may eventually translate into effective and scalable interventions that improve the well-being of children and families.

EST provides one template for understanding how theory-driven work on IPST could be translated into effective, scalable interventions. On the basis of research examining how destructive interparental conflict undermines child emotional security, researchers have developed the Family Communication Project (Cummings & Schatz, 2012), which is a randomized clinical trial designed to improve conflict communication involving both parents and their children. The crux of the intervention relies on a psychoeducational approach: Using activities and examples, couples are educated about conflict communication and are then taught skills to improve their conflict behavior. Multiple studies have shown that this approach is effective in translating the theory-driven, empirical findings of the emotional-security literature into real change for families. Compared with those in control conditions, families randomly assigned to receive psychoeducation experience improvements in key outcomes, such as their child’s emotional security and parent–child attachment security (Cummings & Schatz, 2012; Hoegler et al., 2023; Miller-Graff et al., 2016).

IPST can draw on these successes to determine how findings on interparental positivity might be translated into practical interventions that impact families beneficially. Notably, causal evidence of improvements in relationship outcomes from dyadic studies of positive interpersonal processes has lagged behind that of negative processes, such as addressing marital conflict (e.g., Baucom et al., 1998; Fischer et al., 2016). Nonetheless, that important foundational work attempting to enhance positive interpersonal processes has been underway and should persist (Algoe et al., 2016; Chang et al., 2022; Woods et al., 2015; J. Zhou et al., 2022). Most critically, no prior work has attempted an intervention to enhance positive interpersonal processes in the context of families. We believe that such an intervention could rely on the psychoeducational approach that has been successful in the EST literature.

Although prior research has experimentally manipulated the manner in which people engage in positive processes (in a single gratitude interaction; Algoe et al., 2016), or the extent to which individuals engage in positive interpersonal processes in daily life (Chang et al., 2022), none has adopted the psychoeducation approach, whereby participants are educated and trained (a) to understand the value and importance of these behaviors and (b) how to engage in them effectively.^16^ Like the researchers who developed the Family Communication Project (Cummings & Schatz, 2012), we envision a program in which participants are educated about the importance of positive interactions and are then trained how to engage in these interactions using concrete examples and interactive activities. One reason for optimism for this approach comes from the upward spiral theory of lifestyle change, which suggests that training people to value actions that are infused with positive affect leads more readily to sustained behavioral maintenance than does training them to avoid actions that evoke negative affect (Van Cappellen et al., 2018). This means that a psychoeducational intervention that helps people to fully understand the value of positive relational and familial experiences may be especially likely to be sustained because of the inherently reinforcing nature of positive emotions and positive family interactions.

We believe that the most effective and holistic intervention approach to encouraging the optimal health and development of children should also address both conflict and positive interparental interactions. IPST fully acknowledges the importance of interparental conflict in contributing to child and family dynamics and well-being, which means that interventions aimed at promoting child and family well-being must also address conflict skills. IPST emphasizes, however, that child and family well-being cannot be understood based solely on patterns of conflict communication; positive interactions also generate unique benefits to both child and family well-being. An intervention that addresses both conflict and positive interparental interactions should therefore be most likely to promote healthy child well-being and development. One way to achieve this aim within a single intervention might be to integrate psychoeducational materials relevant to promoting positive interactions with existing, evidenced-based interventions designed to reduce interparental conflict, such as the Family Communication Project.

A final note of caution is warranted. IPST is a theory. Thus, before interventions are designed, rigorous and robust evidence that supports IPST’s core predictions is needed. In addition, encouraging one or both members of a parental couple to change behavior, over time, is a complicated process best informed by interdisciplinary teams, potentially drawing on expertise from social psychology, clinical psychology, developmental psychology, health psychology, and public health (e.g., Chang et al., 2022). Keeping these cautionary notes in mind, the implications for interventions based on IPST are promising and have the potential to impact children and families in many beneficial ways.

## Conclusion

Abundant prior research has documented the importance of interparental relationships on child well-being and development. Despite this, little theory or research has considered whether positive interparental interactions exert a unique, positive impact on children in both the short term and the long term. IPST fills an important gap in the existing literature by explicating how positive interparental interactions are likely to influence their children’s outcomes in beneficial ways, independent of the deleterious effects of interparental conflict. This integrative framework is relevant to work in many areas of psychology, including social, developmental, health, and clinical psychology. In addition, IPST generates several novel and testable predictions. IPST, for example, proposes that to enhance the well-being of children, it is not enough to reduce high levels of negativity within families. Instead, positivity within the parental relationship is needed to promote growth and self-development in a way that is beneficial to children, both during specific moments and across time.

## Transparency

*Action Editor:* Lora Park

*Editor:* Interim Editorial Panel

## Notes

1. Not all positively valenced interactions are necessarily beneficial in terms of their outcomes (McNulty & Fincham, 2012). Although positive interpersonal processes are generally associated with beneficial outcomes, the context of the interaction is crucial, and positively valenced interactions are, at times, associated with maladaptive outcomes depending on the context (McNulty & Fincham, 2012). Our claim here is that positive interpersonal processes generally tend to promote beneficial outcomes for both individuals and couples, consistent with extensive research in affective and relationship science.

2. We note that, within the framework of emotional security theory, researchers have examined the importance of *constructive* behavior during interparental conflict in contributing to child outcomes (e.g., Swerbenski et al., 2023). Likewise, some research in the emotional security theory literature has examined how parent or familial characteristics—such as supportive parenting (DeBoard-Lucas et al., 2010)—lessen the impact of conflict on child outcomes. Crucially, each of these factors has been examined within the context of interparental conflict, meaning little prior research has investigated whether or how positive interparental interactions—interactions that are distinct from conflict—independently influence child well-being and development.

3. Based on theory and prior research in family, relationship, and affective science, interparental positivity spillover theory suggests that these are the three primary ways in which interparental positive interactions spill over into children’s experiences. There are other ways in which children can be affected or influenced by interparental positive interactions. For the purposes of understanding children’s well-being, health, and development, our suggestion here is that these are the most important ways in which interparental positivity influences children.

4. As reviewed by (Clark & Monin, 2014), research has also documented that viewing positive emotions more generally alters our perceptions of and behaviors toward other people, typically in a beneficial manner.

5. One question that arises is whether Jamal might come to view the expresser of the gratitude differently than the recipient in terms of responsiveness. Some research, including the results of Algoe et al. (2020) and other work in the expressed-gratitude literature (Algoe et al., 2013, 2016), suggests a gratitude interaction may be especially likely to influence Jamal’s perceptions of the gratitude expresser. Yet we also suggest that because gratitude expressions typically occur because of a specific good deed that the recipient did, the gratitude expression may also alter Jamal’s view of the responsiveness of the gratitude recipient. Moreover, the positivity-infused interaction more generally is likely to have a beneficial influence on Jamal’s sense that his parents’ relationship is trusting and secure and that he can therefore trust both of them.

6. On the basis of prior work examining the outcomes of one individual’s gratitude expressions on onlookers, we do note that it is possible that one parent expressing positivity (“You’re amazing!”) followed by a neutral response from the partner would likely still produce some degree of positive spillover to the child. Interparental positivity spillover theory simply predicts that parental interactions in which both parents are engaged in the interaction in a warm and active manner are especially likely to positively spill over to influence the child.

7. One reason that people may also feel pressured to experience, express, or pursue positive emotions is beause positive emotions and happiness are highly valued in Western cultures (Bastian et al., 2012, 2015; Ford et al., 2015). This type of pressure to experience and express positive emotions could detrimentally influence the authenticity of interparental positive interactions. We return to the important question of culture later in the article.

8. Another example is when parents engage in excessive shared pleasure-seeking in front of their child. If parents excessively consume alcohol or drugs in front of their child, this may feel subjectively pleasurable to the parents in the moment, but because this form of pleasure-seeking is typically not characterized by shared warmth and mutual care, it is unlikely to result in positivity spillover to the child.

9. Prior research demonstrates that mild, temporary experiences of negative mood can also produce cognitive benefits. Indeed, whereas positive emotions broaden the scope of thought-action repertoires (Fredrickson, 2013), negative mood contributes to more systematic information processing (Fiedler, 2001; Forgas, 2013), which can lead to less gullibility (Forgas & East, 2008), enhanced ability to craft persuasive messages (Forgas, 2007), and memory benefits (Forgas et al., 2009). Thus, although extensive research has documented that positive emotions are associated with cognitive benefits, positive emotions are not universally beneficial to all types of cognitive processing, and temporary experiences of negative mood can play an important role in cognition. Applying these ideas to interparental positivity spillover theory, if Jamal witnesses his parents engage in a gratitude interaction, this should be especially helpful for his cognitive functioning if he has to engage in a creative-writing or problem-solving task (Blau & Klein, 2010; Greene & Noice, 1988). If Jamal needs to detect whether someone is lying, however, witnessing his parents engage in gratitude would not help him (Forgas & East, 2008).

10. One question that arises is whether one of the three subcomponents might contribute to beneficial child social behavior particularly strongly. Because prior work provides reason to expect that each of the three subcomponents of spillover may contribute to children’s positive outcomes, we offer no predictions that any one component is likely to be more or less strongly linked to children’s behavior outcomes in the short term.

11. Many of the long-term outcomes relevant to interparental positivity spillover theory (IPST) are interrelated, including social, mental, and physical well-being. In Figure 2, we do not specify the interconnections between each of these long-term outcomes because (a) this is not the focus of IPST and (b) we chose to maintain the parsimony of the figure, which we felt would become less helpful if we specified every path between all of the outcome variables.

12. We note that positive social interactions typically contain a mixture of discrete positive emotions. Although the discrete emotion of gratitude is a critical part of (and the impetus for) expressed gratitude interactions, people also experience numerous other discrete positive emotions during these interactions, such as joy, amusement, admiration, and love (Algoe et al., 2013, 2016, 2020). In capitalization interactions, too, although shared happiness is the focus, people also experience gratitude, admiration, love, and other discrete positive emotions (Reis et al., 2010). So, although some of the affective content in each of these interactions is unique, much of it is similar, thus supporting a fundamental tenet of IPST—that interparental positive interactions operate as a broad class of behaviors with a shared spillover function.

13. Although we have included contextual factors in Figure 2 only, interparental positivity spillover theory also suggests that contextual factors shape the way in which short-term interactions are experienced by children and their parents. We excluded contextual factors from Figure 1 to simplify the figure.

14. One potential moderating factor is children’s age. In a quantitative summary of literature, van Eldick et al. (2020) found that child age moderated the association between interparental conflict and children’s cognitive appraisals (e.g., self-blame, perceptions of their parents, negative representations of the family), but child age did not moderate the link between interparental conflict and children’s emotional or behavioral outcome. To explain these findings, the authors suggested that, compared with younger children, older children have superior social-cognitive and social-affective skills that allow them to better understand the potential consequences of interparental conflict for themselves and their family. Extrapolating these findings to interparental positivity spillover theory and the three subcomponents of spillover, we suspect that interparental positive interactions will have a stronger impact on older children’s perceptions of their parents but that positive interactions will similarly contribute to the positive emotions and social learning of both older and younger children. We also note that research in developmental psychology demonstrates that the perceptions and behavior of 4- and 5-year-old children are influenced by observing other people express gratitude, although this is truer among 5-year-old children (Vaish & Savell, 2022). Thus, although older children have superior social-cognitive and social-affective skills compared with younger ones, younger children are still able to recognize and be influenced by other people’s positive interpersonal actions.

15. The link between coparenting and positive interparental interactions is likely bidirectional: Healthy coparenting likely contributes to positive interparental interactions, and positive interparental interactions most likely contribute to enhanced coparenting, possibilities that future research should test.

16. One exception is a study by Woods et al. (2015), in which participants received a short training on how to respond to partners’ capitalization attempts in an active-constructive manner.
